# Association between Metabolic Syndrome Score and Subclinical Atherosclerosis

**DOI:** 10.31083/RCM26811

**Published:** 2025-03-12

**Authors:** Ming Yi, Xinyi Wang, Yan Li, Xuewen Li, Jin Si, Yinghua Zhang, Keling Xiao, Lijie Sun, Haoyu Zhang, Jinghao Sun, Zhaoli Liu, Jiaying Lin, Yuxin Xie, Bingyan Zhang, Jing Zhao, Xi Chu, Jing Li

**Affiliations:** ^1^Department of Geriatrics, Xuanwu Hospital, Capital Medical University, National Clinical Research Center for Geriatric Diseases, 100053 Beijing, China; ^2^Department of the General Medicine, Characteristic Medical Center of Chinese People’s Armed Police Force, 300162 Tianjin, China; ^3^Department of Cardiology, Chui Yang Liu Hospital Affiliated to Tsinghua University, 100021 Beijing, China; ^4^Health Management Center, Xuanwu Hospital, Capital Medical University, 100053 Beijing, China

**Keywords:** metabolic syndrome score, pulse wave velocity, flow-mediated dilation, arterial stiffness, endothelial dysfunction, subclinical atherosclerosis

## Abstract

**Background:**

Previous studies have presented conflicting results on the correlation between metabolic syndrome (MetS) and subclinical atherosclerosis. However, the binary MetS definition cannot reflect the severity of metabolic disorders continuously and dynamically. The present study calculated the MetS score and explored the association between MetS score and subclinical atherosclerosis.

**Methods:**

A total of 840 participants were included in this observational, cross-sectional study; 66.55% of participants were men, and the median age was 61.00 years (53.00, 67.00). Brachial–ankle pulse wave velocity (baPWV) and brachial flow-mediated dilation (bFMD) values were measured from October 2016 to January 2020. Spearman’s correlation and multiple linear regression analyses were conducted to explore the correlation between the MetS score and baPWV and bFMD. Arterial stiffness was defined as baPWV ≥1400 cm/s, while endothelial dysfunction was described as bFMD >6%. Multiple logistic regression was performed to explore the effects of MetS and MetS score on arterial stiffness and endothelial dysfunction.

**Results:**

The MetS score was significantly associated with baPWV (β = 73.59, 95% CI (42.70, 104.48); *p* < 0.001) and bFMD (β = –0.43, 95% CI (–0.75, –0.10); *p* = 0.010) after adjusting for covariates. Compared with the binary definition of MetS, the MetS score was a more significant predictor for arterial stiffness (odds ratio, OR = 2.63, 95% CI (1.85, 3.74); *p* < 0.001) and endothelial dysfunction (OR = 1.33, 95% CI (1.01, 1.76); *p* = 0.040). Leukocyte count (r = 0.32; *p* < 0.001) and high-sensitivity C-reactive protein (hs-CRP) (r = 0.17; *p* < 0.001) values were related to the MetS score.

**Conclusions:**

The MetS score is a clinically accessible assessment of metabolic status that can identify individuals at higher risk of subclinical atherosclerosis.

## 1. Introduction

The prevalence of metabolic syndrome (MetS), a constellation of metabolic disorders including hypertension, glucose intolerance, dyslipidemia, and central obesity, is increasing worldwide [1, 2, 3]. Moreover, MetS was found to correlate with chronic inflammation, oxidative stress, and prothrombotic state, contributing to vascular wall remodeling and stiffening [4, 5, 6]. Well-documented studies have established that MetS is an independent risk factor for cardiovascular diseases (CVDs) [6, 7]. However, several studies found that MetS does not exhibit any additional predictive ability beyond other risk assessments, such as the Framingham Risk Score [8, 9]. Furthermore, conflicting evidence exists on whether MetS is associated with subclinical atherosclerosis [10], chronic kidney disease (CKD) [11, 12], and all-cause mortality [13, 14]. Indeed, previous studies have been limited by the binary nature of the MetS definition, which does not reflect the continuous severity of metabolic disorders; thus, a large part of the required information may be omitted [15]. To address this limitation, recent studies have developed MetS score to assess individuals’ metabolic status continuously [16, 17, 18, 19, 20, 21]. Subsequently, these studies found that MetS score was related to CVD [19], all-cause mortality [19], diabetes mellitus (DM) [22], and CKD [21, 23].

Subclinical atherosclerosis is an early detectable manifestation of atherosclerosis before clinical symptoms appear. Brachial–ankle pulse wave velocity (baPWV) and brachial flow-mediated dilation (bFMD) were widely accepted as subclinical atherosclerosis markers since they are simple, noninvasive, and radiation-free [24, 25]. BaPWV is a simple and repeatable technique to assess arterial stiffness by calculating the wave transmission velocity between the recording sites on the brachial and posterior tibial arteries [26]. Additionally, bFMD evaluates endothelial function by examining brachial artery diameter changes in response to ischemia [27]. Previous studies found that each standard deviation unit increase in the baPWV was associated with a 20% higher risk of CVD. In comparison, a 1% decrease in the bFMD correlated with a 13% increased risk of CVD [28, 29].

Although several studies based on the binary MetS definition have investigated the correlation between MetS and subclinical atherosclerosis [30, 31, 32, 33], whether the newly developed MetS score correlates with subclinical atherosclerosis remains to be confirmed. Therefore, the current study calculated the MetS score and aimed to explore the association between it and subclinical atherosclerosis assessed using the baPWV and bFMD.

## 2. Materials and Methods

### 2.1 Study Population

Participants who had regular physical examinations at the Health Management Department of Xuanwu Hospital, Capital Medical University, along with individuals with a history of CVD who regularly visited the Outpatient Department of Xuanwu Hospital between October 2016 and January 2020, were included in the study. Information, including demographics, lifestyle, medical history, and physical and biochemical measurements, was collected from the electronic medical record system. Written informed consent was obtained from all participants. The study protocol was approved by the Ethics Committee of the Xuanwu Hospital, Capital Medical University.

### 2.2 Physical and Biochemical Measurements

The current study collected demographic data, lifestyle information, and medical history from participants’ medical records at Xuanwu Hospital Capital Medical University. Blood pressure (BP) was measured using an electronic sphygmomanometer in the right arm, with each subject sitting for at least 10 minutes. Mean arterial blood pressure (MAP) was calculated as ((2 × diastolic blood pressure) + systolic blood pressure)/3. Waist circumference (WC) was measured at the umbilical level using a flexible steel tape and measured to the nearest millimeter. Participants with self-reported medical histories or those receiving specific drug treatments were considered as having developed hypertension or type 2 diabetes mellitus (T2DM). Hypercholesterolemia was defined as either total cholesterol (TC) ≥6.2 mmol/L, a self-reported or physician diagnosis, or the use of lipid-lowering medication [34]. Central obesity was defined as a WC ≥90 cm in males and ≥85 cm in females. Blood samples were obtained via venipuncture after participants had fasted for at least 8 hours. TC and triglycerides (TGs) were measured using enzymatic methods. High-density lipoprotein cholesterol (HDL-C) was measured by direct testing methods after dextran–magnesium precipitation. Low-density lipoprotein cholesterol (LDL-C) was calculated using the Friedewald equation. Fasting blood glucose (FBG) was measured using the hexokinase method. MetS was defined using five components, including WC, FBG, MAP, and HDL-C and TG levels, according to the National Cholesterol Education Program Adult Treatment Panel III [35].

### 2.3 Metabolic Syndrome Score Calculation

The current study used principal component analysis (PCA) with varimax rotation to formulate the MetS scores from five components of traditional MetS definition (WC, FBG, MAP, and HDL-C and TG levels). The reciprocal of HDL-C was applied since HDL-C negatively correlates with metabolic risk. A previously reported calculation method was employed [17, 19, 21]. Briefly, two principal components (PCs) were derived from the WC, MAP, FBG, TG, and the reciprocal of HDL-C values to represent the majority of the variance (eigenvalue ≥1.0). PC1 and PC2 explained 28.04% and 27.96% of the variance in the present study. Five MetS components were standardized before calculation. The weights (PC1, PC2) of each component were: WC (–0.06, 0.58), MAP (–0.18, 0.60), FBG (0.53, –0.19), TG (0.57, –0.08), and HDL-C reciprocal (0.36, 0.20). Then, individual PC scores were summed to generate the PC1 and PC2. Finally, the MetS score was calculated as 28.04% × PC1 + 27.96% × PC2. A higher MetS score indicates a less favorable metabolic profile.

### 2.4 BaPWV Measurement

The present study measured the baPWV using an automatic waveform analyzer with appropriate-sized cuffs (BP-203RPEIII, OMRON HEALTHCARE Co., Ltd., Kyoto, Japan). After resting for at least 10 minutes in the supine position, occlusion and monitoring cuffs were wrapped around both sides of the upper arms and ankles to measure baPWV. The baPWV was calculated as the distance between brachial and posterior tibial arterial recording sites divided by transmission time [26]. The maximum value of the left and right baPWV was used in the statistical analyses. The baPWV cutoff value for diagnosing arterial stiffness was 1400 cm/s [29].

### 2.5 bFMD Measurements

A B-mode ultrasound image with a 7.5 MHz linear array transducer (UNEX-EF, UNEX Co., Ltd., Nagoya, Japan) was used to measure vasodilator responses in the brachial arteries. Brachial artery scanning was performed longitudinally within the antecubital fossa. After baseline measurements of the brachial artery diameter, a blood pressure cuff was placed around the forearm, inflated to a pressure exceeding systolic blood pressure with >50 mmHg for five minutes, and then released. Diameter measurements during reactive hyperemia were recorded for three minutes after cuff deflation. The bFMD was calculated as the percentage change in peak vessel diameter from baseline ((peak diameter - baseline diameter)/baseline diameter) [27]. A bFMD value <6% was defined as endothelial dysfunction [25].

### 2.6 Statistical Analysis

Continuous variables are presented as the mean ± standard deviation (SD) or median (interquartile range) for skewed variables. Categorical variables are presented as numbers (percentages). Spearman’s correlation analyses evaluated the correlation between the MetS score and baPWV, bFMD, leukocyte, and high-sensitivity C-reactive protein (hs-CRP) values. Participants with missing hs-CRP values were excluded from the analysis. Multiple linear regression analyses explored the linear relation between the MetS score and baPWV and bFMD. Multiple logistic regression was performed to explore whether MetS and MetS score are independent risk factors for arterial stiffness and endothelial dysfunction. Receiver operating characteristic (ROC) analyses were also performed to compare the ability of MetS and MetS scores to predict arterial stiffness and endothelial dysfunction. Baseline variables considered clinically relevant or with *p*-values of less than 0.10 in the univariate analysis were entered into the models while avoiding multicollinearity and overadjustment. The following covariates were included in all analyses: age, sex, smoking history, history of coronary heart disease (CHD), hypertension, DM, hypercholesterolemia, and central obesity. A two-sided *p*-value < 0.05 was considered statistically significant. All statistical analyses were performed using SPSS version 25.0 (SPSS Inc., Chicago, IL, USA).

## 3. Results

**Fig. 1.**
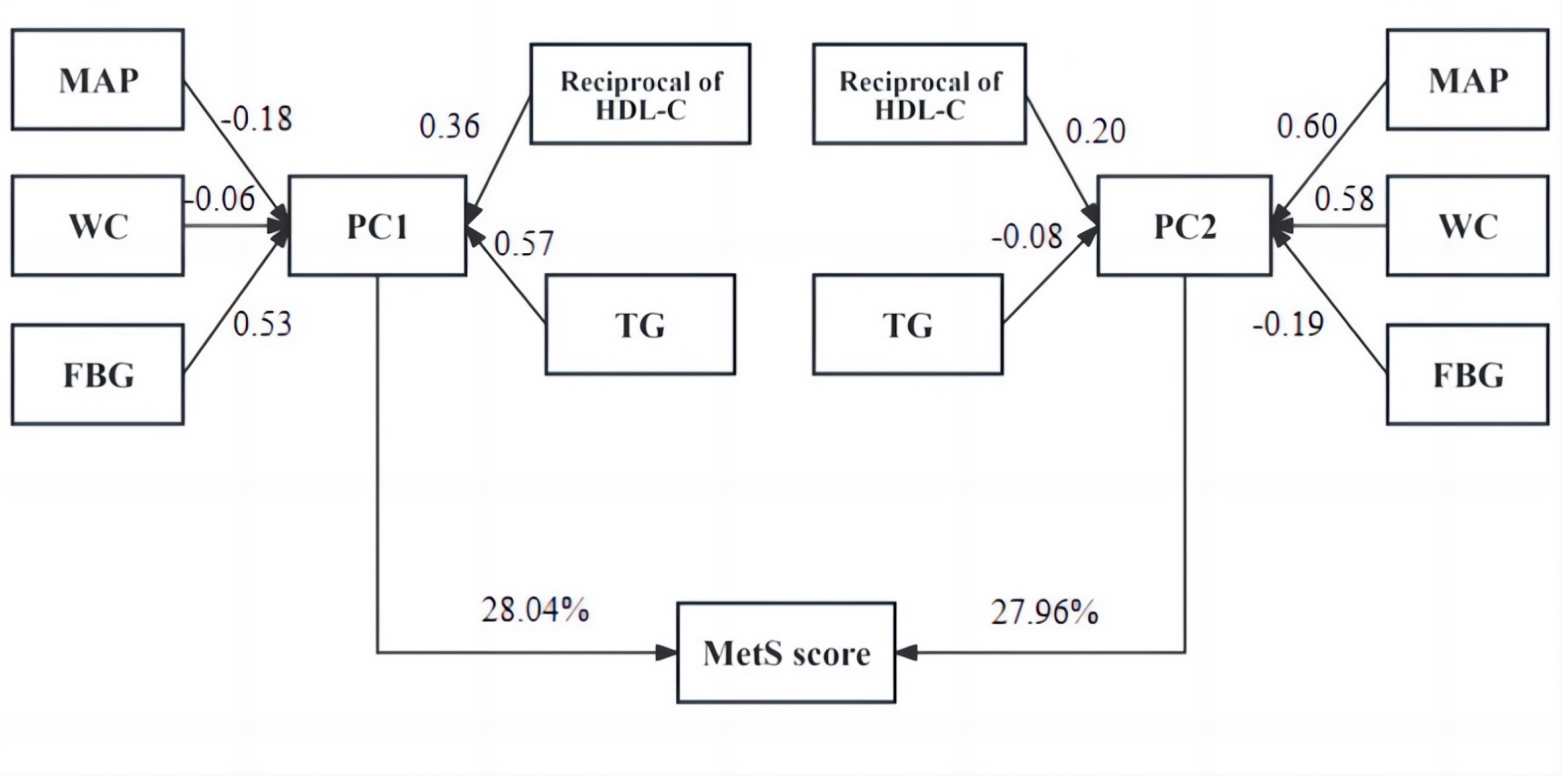
**MetS score calculation procedure from MAP, WC, FBG, HDL-C, and TG values**. Abbreviations: WC, waist circumference; PC, principal component.

In total, 840 participants were included in the cross-sectional study from October 2016 to January 2020. The demographic and clinical characteristics of participants are shown in Table 1. The median age of study participants was 61.00 (53.00, 67.00) years, and 559 (66.55%) were male. Moreover, 47.62% of participants had a history of smoking. The proportions for participants with CHD, hypertension, DM, hypercholesterolemia, and central obesity were 51.19%, 58.10%, 29.17%, 38.93%, and 63.21%, respectively. Furthermore, 51.55% of participants were diagnosed with MetS. The MetS score was normally distributed from –2.04 to 2.81. The mean MetS score value was 0 ± 0.71. The median MetS score value was 0.04 (–0.45, 0.43). The median baPWV and bFMD values were 1528.50 cm/s (1385.25, 1733.50) and 5.10% (3.40, 7.00), respectively. Fig. 1 shows the principal component analysis results and the MetS score calculation procedure.

**Table 1.** **Demographic and clinical characteristics of participants**.

Variables	Total
	(n = 840)
Age (years)	61.00 (53.00, 67.00)
Males, n (%)	559 (66.55)
Smoking history, n (%)	400 (47.62)
History of CHD, n (%)	430 (51.19)
Hypertension, n (%)	488 (58.10)
DM, n (%)	245 (29.17)
Hypercholesterolemia, n (%)	327 (38.93)
Central obesity, n (%)	531 (63.21)
MetS, n (%)	433 (51.55)
SBP (mmHg)	130.00 (119.00, 142.00)
DBP (mmHg)	79.00 (71.00, 87.00)
MAP (mmHg)	95.67 (88.67, 104.00)
Serum uric acid (µmol/L)	339.00 (284.00, 398.00)
Serum creatinine (µmol/L)	68.00 (57.00, 77.00)
FBG (mmol/L)	5.25 (4.80, 6.35)
TC (mmol/L)	4.12 (3.53. 4.90)
TG (mmol/L)	1.48 (1.08, 2.15)
LDL-C (mmol/L)	2.45 (1.98, 3.04)
HDL-C (mmol/L)	1.10 (0.92, 1.34)
White blood cell (×10^9^/L)	6.29 (5.21, 7.62)
hs-CRP (mg/L)	1.47 (0.56, 3.41)
baPWV (cm/s)	1528.50 (1385.25, 1733.50)
bFMD (%)	5.10 (3.40, 7.00)
MetS score	0 ± 0.71

Abbreviations: CHD, coronary heart diseases; DM, diabetes mellitus; MetS, metabolic syndrome; SBP, systolic blood pressure; DBP, diastolic blood pressure; MAP, mean arterial blood pressure; FBG, fasting blood glucose; TC, total cholesterol; TG, triglyceride; LDL-C, low-density lipoprotein cholesterol; HDL-C, high-density lipoprotein cholesterol; hs-CRP, high-sensitivity C-reactive protein; baPWV, brachial–ankle pulse wave velocity; bFMD, brachial flow-mediated dilation.

**Fig. 2.**
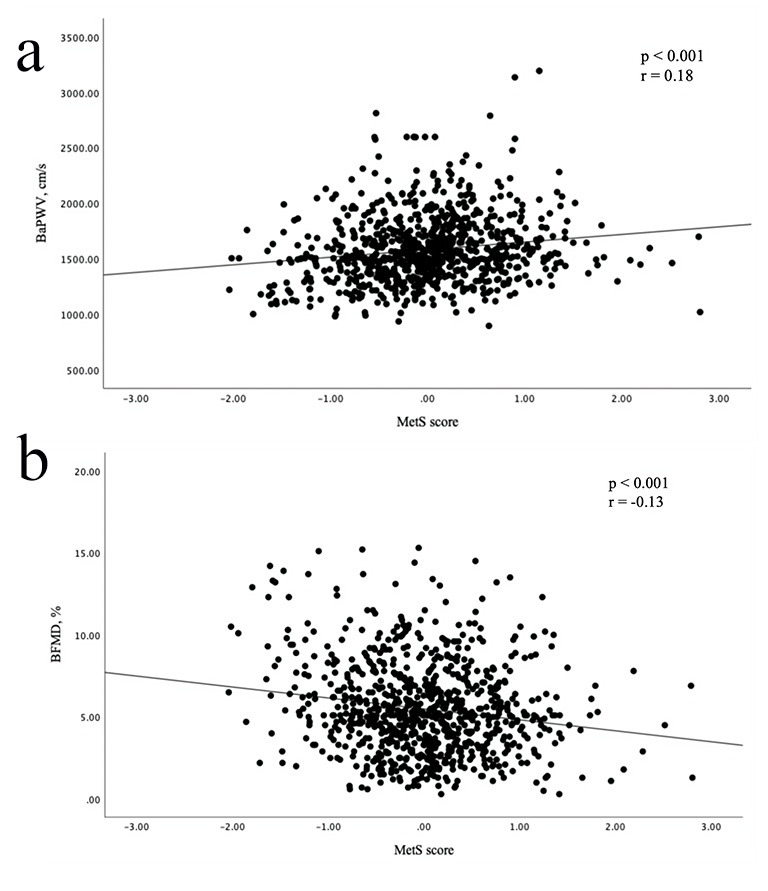
**Scatter plots showing correlations between MetS score and baPWV and bFMD**. (a) MetS score was positively correlated with baPWV. (b) MetS score was inversely associated with bFMD.

As described in Fig. 2, Spearman’s correlation analysis showed that the MetS score was positively correlated with baPWV (r = 0.18; *p* < 0.001) and inversely correlated with bFMD (r = –0.13; *p* < 0.001). In the multiple linear analysis shown in Table 2, we found that each one-unit MetS score increment was associated with a 73.59 cm/s (95% CI (42.70, 104.48); *p* < 0.001) increase in baPWV value and a 0.43% (95% CI (–0.75, –0.10); *p* = 0.010) decrease in bFMD value after adjusting for age, sex, smoking history, history of hypertension, CHD, DM, hypercholesterolemia, and central obesity.

**Table 2.** **Multiple linear regression model for MetS score and baPWV, bFMD**.

	baPWV		bFMD	
Variables	β (95% CI)	*p* value	β (95% CI)	*p* value
Age (years)	14.74 (12.95, 16.52)	<0.001	–0.06 (–0.08, –0.05)	<0.001
Sex (Female, %)	13.04 (–33.03, 59.11)	0.579	0.20 (–0.29, 0.68)	0.425
Smoking history (%)	–5.82 (–48.02, 36.39)	0.787	–0.53 (–0.98, –0.09)	0.019
CHD (%)	25.21 (–15.42, 65.84)	0.224	–0.15 (–0.58, 0.28)	0.498
Hypertension (%)	41.84 (4.38, 79.29)	0.029	–0.56 (–0.95, –0.16)	0.006
Diabetes mellitus (%)	49.43 (8.06, 90.80)	0.019	–0.25 (–0.69, 0.18)	0.253
Central obesity (%)	5.79 (–12.07, 23.65)	0.525	0.08 (–0.11, 0.26)	0.429
Hypercholesterolemia (%)	–36.30 (–76.15, 3.56)	0.074	0.12 (–0.30, 0.54)	0.571
MetS score	73.59 (42.70, 104.48)	<0.001	–0.43 (–0.75, –0.10)	0.010

**Fig. 3.**
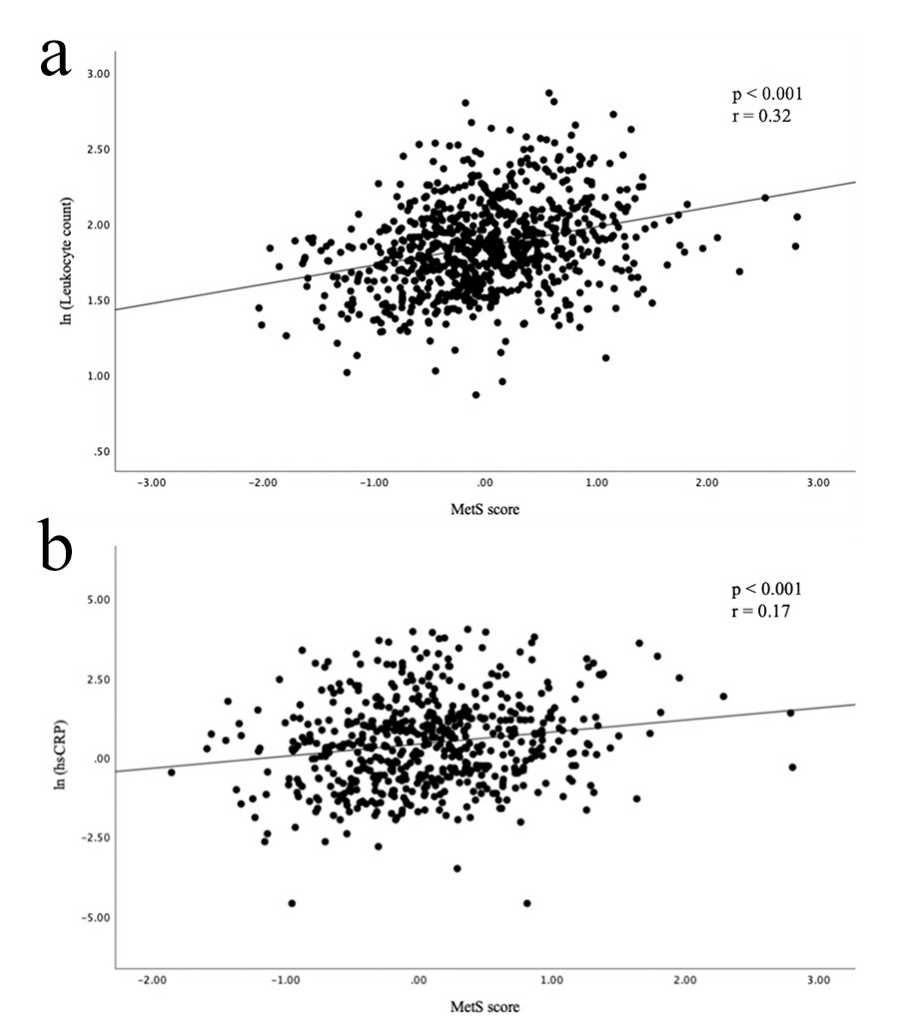
**Scatter plots showing a correlation between MetS score and inflammatory markers**. (a) MetS score was positively correlated with leukocyte count. (b) MetS score was positively correlated with hs-CRP.

The baPWV and bFMD values were used to define arterial stiffness and endothelial dysfunction. In the multiple logistic regression analysis shown in Table 3, the binary MetS definition was not an independent risk factor for arterial stiffness and endothelial dysfunction. Comparatively, the MetS score was a significant predictor for arterial stiffness (odds ratio, OR = 2.63, 95% CI (1.85, 3.74); *p* < 0.001) and endothelial dysfunction (OR = 1.33, 95% CI (1.01, 1.76); *p* = 0.040). Further comparsion between MetS score and MetS were depicted with ROC curves in **Supplementary Fig. 1** and **Supplementary Fig. 2**. In the ROC curve analysis shown in **Supplementary Table 1**, the area under ROC curve (AUC) of MetS and the MetS score for arterial stiffness were 0.570 (95% CI (0.526, 0.613); *p* = 0.002) and 0.612 (95% CI (0.569, 0.655); *p* < 0.001), respectively. In the ROC curve analysis shown in **Supplementary Table 2**, the AUC values for MetS and the MetS score for endothelial dysfunction were 0.561 (95% CI (0.521, 0.601); *p* = 0.003) and 0.571 (95% CI (0.530, 0.612); *p* = 0.001), respectively. Fig. 3 shows the leukocyte count (r = 0.32; *p* < 0.001) and hs-CRP value (r = 0.17; *p* < 0.001) were significantly correlated with the MetS score.

**Table 3.** **Multiple logistic regression model for arterial stiffness and endothelial dysfunction**.

	Arterial stiffness		Endothelial dysfunction	
Scoring system	OR (95% CI)	*p*-value	OR (95% CI)	*p*-value
MetS	1.47 (0.93, 2.31)	0.098	1.38 (0.93, 2.03)	0.106
MetS score	2.63 (1.85, 3.74)	<0.001	1.33 (1.01, 1.76)	0.040

Abbreviations: OR, odds ratio. All models were adjusted for age, sex, smoking history, history of coronary heart disease, hypertension, diabetes mellitus, hypercholesterolemia, and central obesity.

## 4. Discussion

In total, 840 participants were included in this cross-sectional study from October 2016 to January 2020, and we calculated the MetS scores from WC, MAP, FBG, and TG and HDL-C levels. The baPWV and bFMD were measured in all participants to assess subclinical atherosclerosis. After adjusting for covariates, we found that the MetS score was significantly associated with baPWV and bFMD. Compared with the dichotomous MetS definition, the continuous MetS score was a more significant predictor for arterial stiffness and endothelial dysfunction. Moreover, inflammatory biomarkers were positively correlated with the MetS score. To our knowledge, this is the first study exploring the correlation between the MetS score and subclinical atherosclerosis.

MetS was found to be correlated with a nearly two-fold increased risk for CVD [36] and has become a global public health challenge [37]. However, the traditional diagnostic criteria of MetS had several limitations. Firstly, the MetS definition has a binary nature (yes or no) that cannot continuously reflect the severity of metabolic disorders. As a result, patients were not fully informed of their metabolic status, and the dynamic change could not be assessed over time. Secondly, the MetS definition only identifies risk when an individual exhibits abnormalities beyond the cutoff values for three of five MetS components. Thus, individuals with deteriorating metabolic status cannot be detected early. To address these limitations, Katrien Wijndaele *et al*. [17] applied PCA to calculate the MetS score as a continuous tool to assess individuals’ metabolic status and found that the MetS score was highly correlated with cardiovascular risk factors. Using the same method to calculate the MetS score, recent studies found that the MetS score was an independent predictor for multiple diseases. Tang *et al*. [19] found each SD unit increase in MetS score contributed to 1.36-fold and 1.16-fold increased risk for CVD and all-cause mortality in healthy participants. Wu *et al*. [21] reported each unit increase in MetS score was related to a 30% increased risk for CKD.

The development of atherosclerosis could start at an early age and progress silently, ultimately resulting in angina, myocardial infarction, or ischemic stroke [38]. The baPWV and bFMD are noninvasive and accessible measurements for subclinical atherosclerosis. Furthermore, the validity and reproducibility of baPWV and bFMD have been confirmed in previous studies [39, 40]. Measured simply by wrapping pressure cuffs around four extremities, baPWV has been validated as a reliable marker for arterial stiffness [29]. Meanwhile, endothelial dysfunction is an essential step in the development and progression of atherosclerosis [41]. Measuring bFMD as an index of endothelium-dependent vasodilation is useful for assessing endothelial function [27]. Impaired baPWV and bFMD have been demonstrated to predict adverse cardiovascular events and worse cardiovascular outcomes, showing great value in primary and secondary prevention strategies for CVD [28, 42]. Nonetheless, the association between the MetS score and subclinical atherosclerosis has yet to be elucidated.

Previous studies found that MetS was an independent predictor for arterial stiffness. For example, Chen *et al*. [30] assessed 8599 Chinese participants and found that baPWV was significantly higher in individuals with MetS. Another study involving 20,570 participants from eight European countries and the US found a positive correlation between MetS and carotid–femoral PWV [43]. Moreover, additional studies found that baPWV increased progressively with the increasing number of abnormal MetS components [44, 45, 46]. The Asymptomatic Polyvascular Abnormalities in Community (APAC) study found the number of MetS components was positively correlated with baPWV in 5181 Chinese participants [44]. A previous meta-analysis including 32 cross-sectional articles found the pooled effect size for arterial stiffness nearly doubled as the number of MetS components increased, from 0.11 for one MetS component to 0.26 for two and 0.4 for three or more [45]. However, the binary MetS definition cannot detect such differences since it only identifies risk when individuals exhibit more than three abnormal MetS components. Previous studies [19, 20, 21, 22, 23, 44, 45, 46] indicated a continuous spectrum of risk underlying the components of MetS, eliminating the need to dichotomize it. However, counting the number of abnormal MetS components does not consider the different weighting of each component in relation to baPWV. Compared with previous studies, the current study applied the MetS score to assess the severity of metabolic disorders and found that the MetS score was a better predictor for arterial stiffness than the binary MetS definition.

Previous studies based on the definition of dichotomous MetS showed conflicting results regarding the correlation between MetS and endothelial dysfunction. Lind [47] performed bFMD measurements in 1016 participants aged 70 years and found no difference in bFMD between participants with and without MetS. The Firemen and Their Endothelium (FATE) study, a large cohort study including 1417 middle-aged participants without CVD and DM, found no association between MetS and bFMD [48]. By contrast, de Matthaeis *et al*. [49] found individuals with MetS were more likely to exhibit endothelial dysfunction in 80 participants with a mean age of 70.3 years. Another study with a larger study population found MetS was related to lower bFMD values in 2123 participants without CVD and DM [50]. The current study showed that continuous MetS scores, not the binary MetS definition, were an independent risk factor for endothelial dysfunction assessed by bFMD. One possible explanation for this paradox is that the correlation between MetS and endothelial function was underestimated in previous studies since the traditional definition of MetS dichotomizes continuous variables for diagnosis; thus, a large part of the information was missed.

Furthermore, our study found inflammatory biomarkers, including leukocyte count and hs-CRP, positively related to MetS score. These findings align with several studies that found individuals with MetS have higher levels of multiple inflammatory biomarkers [51, 52, 53, 54]. As previous studies have established the essential role of inflammation in the development and progression of atherosclerosis [4, 55, 56], individuals with MetS were likely under chronic exposure to an inflammatory state that induced subclinical atherosclerosis.

Using the continuous MetS score has several strengths. Firstly, the data used to calculate the MetS score can easily be collected in the routine physical examination. Therefore, calculating and applying the MetS score will not incur additional costs. Secondly, the MetS score is a continuous assessment of metabolic status, enabling patients to track the dynamic changes over time. Thirdly, the MetS score is an independent predictor for subclinical atherosclerosis, thus improving patients’ self-awareness of their metabolic status and compliance with medical advice.

However, there are also some potential limitations in the present study. First, our cross-sectional study cannot examine the validity of the MetS score in predicting the progression of subclinical atherosclerosis. Second, only 575 participants presented complete records for hs-CRP readings. Therefore, future studies with larger sample sizes must confirm the relationship between the MetS score and inflammatory biomarkers. Third, various parameters, including carotid plaque and coronary artery calcium, can be used to assess subclinical atherosclerosis. Further studies are warranted to investigate the association between the MetS score and other subclinical atherosclerosis parameters and cardiovascular events.

## 5. Conclusions

In conclusion, the current study applied the MetS score to assess the continuous severity of metabolic disorders and found that the MetS score was an independent predictor of arterial stiffness and endothelial dysfunction. The MetS score is an inexpensive and clinically accessible tool that can assess the metabolic status of individuals and identify individuals at higher risk of subclinical atherosclerosis at an early stage.

## Data Availability

The data that support the findings of this study are available on request from the corresponding author.

## Abbreviations

AUC, area under ROC curve; baPWV, brachial-ankle pulse wave velocity; bFMD, brachial flow-mediated dilation; CHD, coronary heart disease; CI, confidence interval; CKD, chronic kidney disease; CVD, cardiovascular diseases; DBP, diastolic blood pressure; DM, diabetes mellitus; FBG, fasting blood glucose; ROC, receiver operating characteristic; HDL-C, high-density lipoprotein cholesterol; hs-CRP, high-sensitivity C-reactive protein; LDL-C, low-density lipoprotein-cholesterol; MAP, mean arterial blood pressure; MetS, metabolic syndrome; OR, odds ratio; PC, principal component; SBP, systolic blood pressure; SD, standard deviation; TC, total cholesterol; TG, triglyceride; WC, waist circumference.

## Supplementary Material

Supplementary material associated with this article can be found, in the online version, at https://doi.org/10.31083/RCM26811.
