# Supplementary material for: Association between Metabolic Syndrome Score and Subclinical Atherosclerosis
Source: Rev Cardiovasc Med. 2025 Mar 12;26(3):26811. doi: 10.31083/RCM26811 (PMC11951480; doi:10.31083/RCM26811)
Supplement: Supplementary file 1 [file 2153-8174-26-3-26811-s1.zip › Supplementary material.docx]

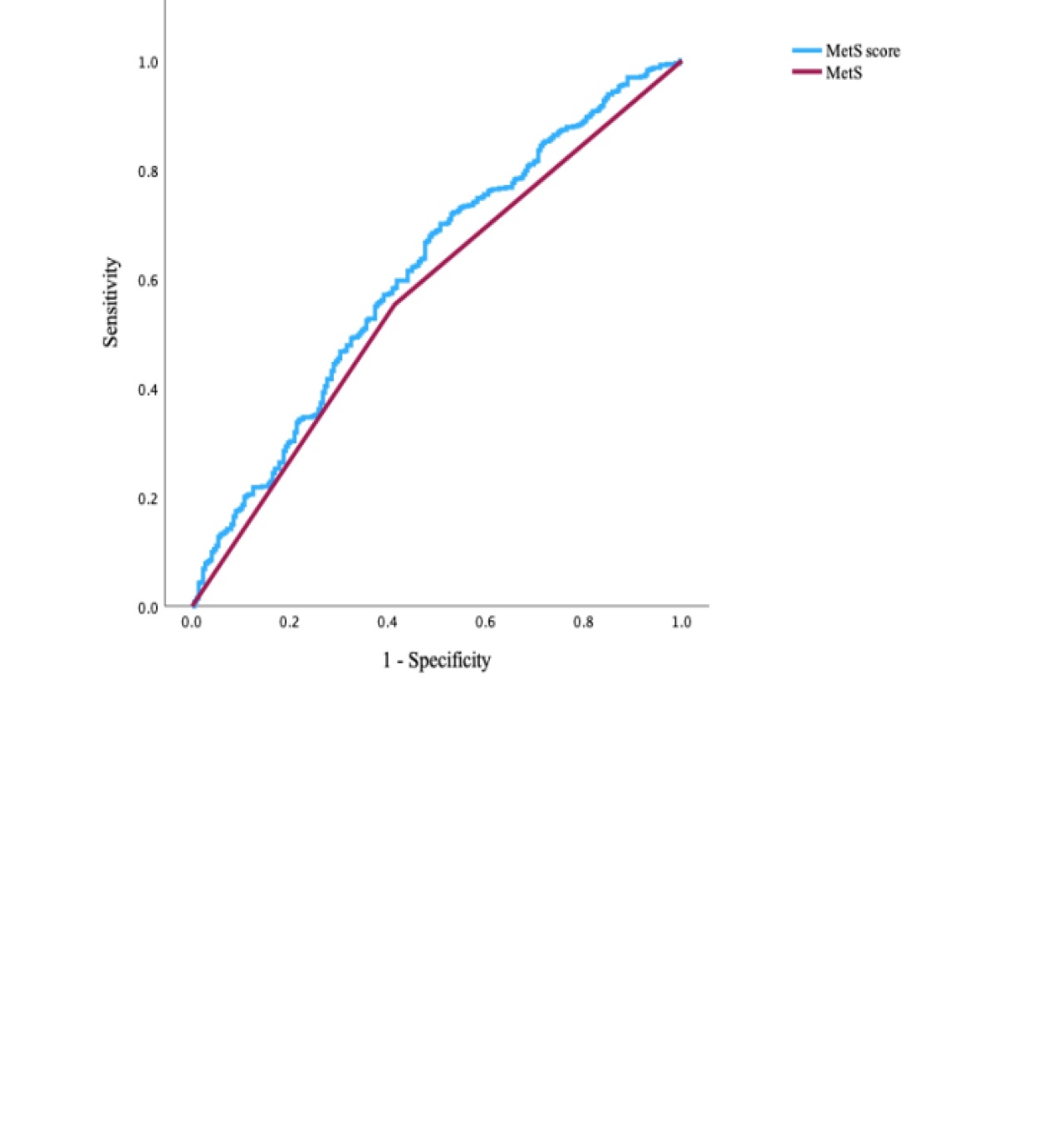


**Supplementary Fig. 1. ROC curve analysis for arterial stiffness.** Abbreviations: ROC, receiver operating characteristic; MetS, metabolic syndrome.


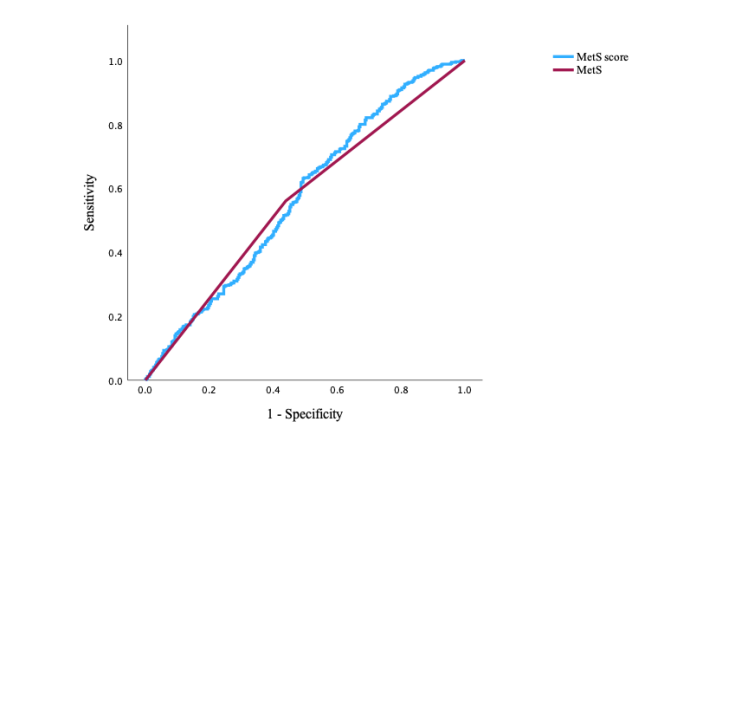


**Supplementary Fig. 2. ROC curve analysis for endothelial dysfunction.** Abbreviations: ROC, receiver operating characteristic; MetS, metabolic syndrome.

Supplementary Table 1. ROC analysis results of MetS, MetS score for arterial stiffness.

| Models | AUC | 95% CI | Threshold | Sensitivity | Specificity |
| --- | --- | --- | --- | --- | --- |
| Mets | 0.570 | (0.526, 0.613) | 0.5 | 55.3 | 58.7 |
| MetS score | 0.612 | (0.569, 0.655) | –0.219 | 68.3 | 51.1 |

Abbreviations: ROC, receiver operating characteristic; AUC, area under ROC curve; CI, confidence interval; MetS, metabolic syndrome.

Supplementary Table 2. ROC analysis results of MetS, MetS score for endothelial dysfunction.

| Models | AUC | 95% CI | Threshold | Sensitivity | Specificity |
| --- | --- | --- | --- | --- | --- |
| MetS | 0.561 | (0.521, 0.601) | 0.5 | 56.1 | 56.1 |
| Mets score | 0.571 | (0.530, 0.612) | –0.138 | 63.1 | 50.6 |

Abbreviations: ROC, receiver operating characteristic; AUC, area under ROC curve; CI, confidence interval; MetS, metabolic syndrome.
