# Supplementary figures and images for: Association between Metabolic Syndrome Score and Subclinical Atherosclerosis
Source: Rev Cardiovasc Med. 2025 Mar 12;26(3):26811. doi: 10.31083/RCM26811 (PMC11951480; doi:10.31083/RCM26811)

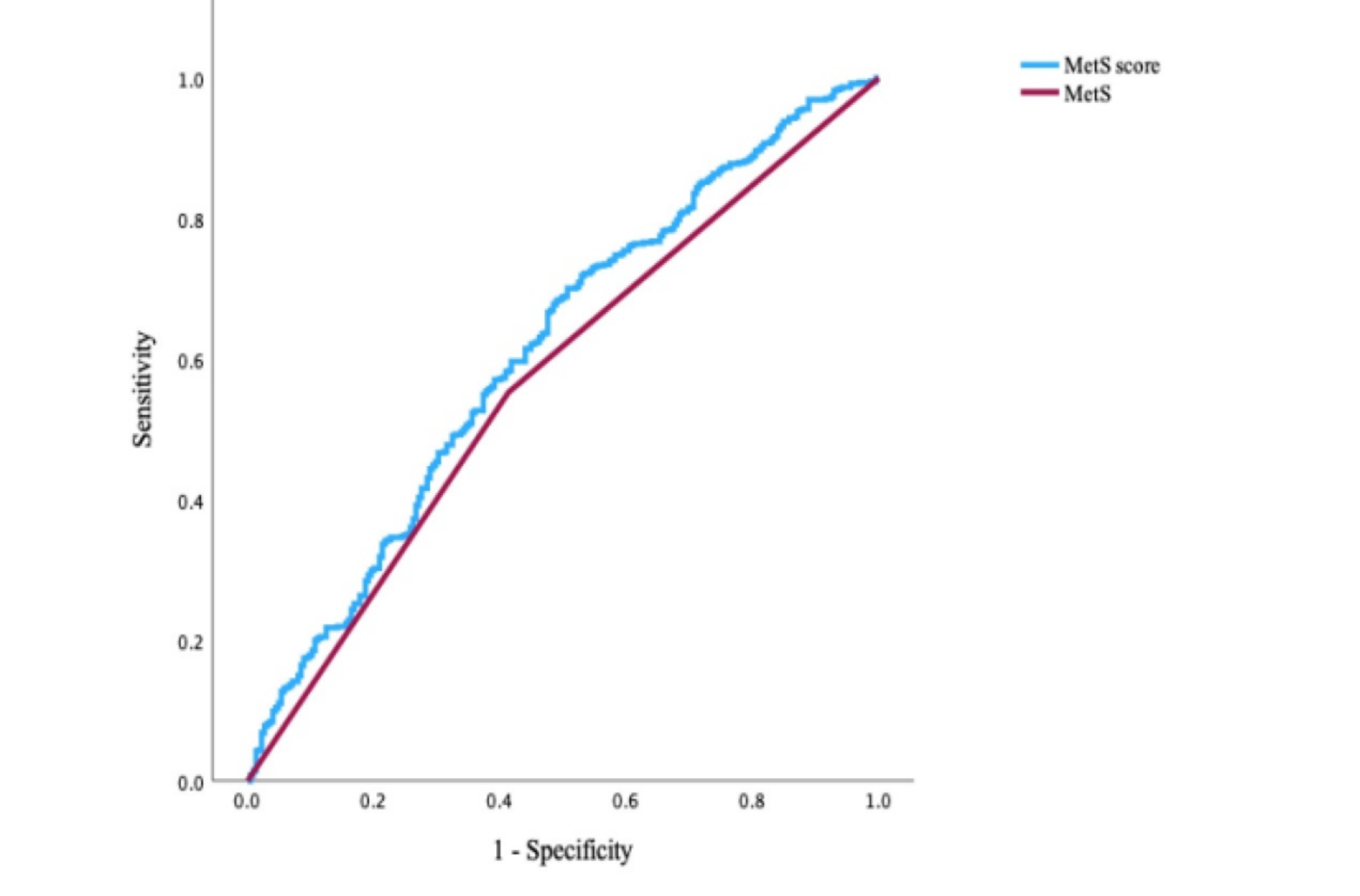

Supplement: Supplementary file 1 [file 2153-8174-26-3-26811-s1.zip › Supplementary+Figure+1.png]

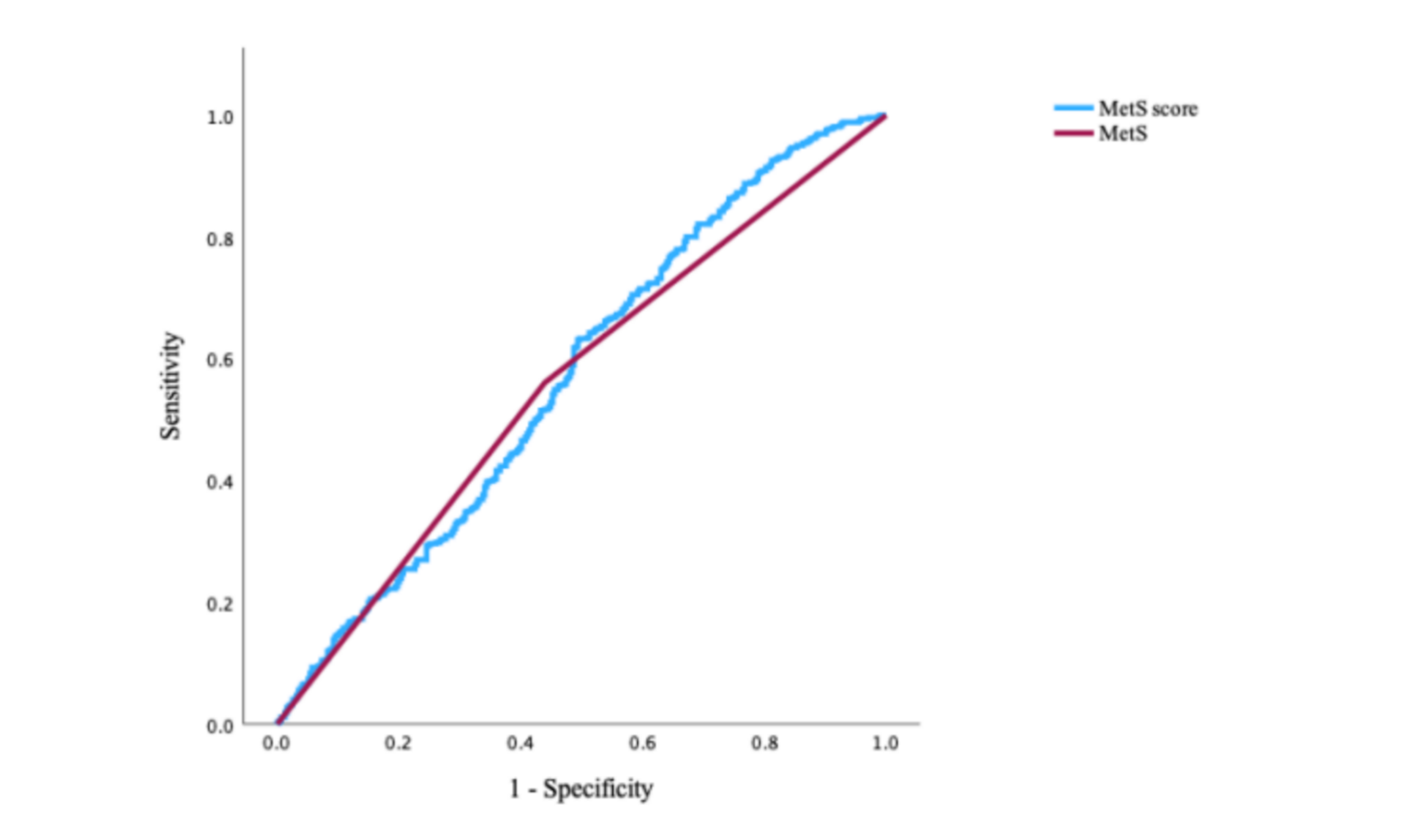

Supplement: Supplementary file 1 [file 2153-8174-26-3-26811-s1.zip › Supplementary+Figure+2.png]
